# Role of Mono- and Disaccharide Combination in Cryoprotective Medium for Rooster Semen to Ensure Cryoresistance of Spermatozoa

**DOI:** 10.3390/molecules26195920

**Published:** 2021-09-29

**Authors:** Olga Stanishevskaya, Yulia Silyukova, Nikolai Pleshanov, Anton Kurochkin

**Affiliations:** Russian Research Institute of Farm Animal Genetics and Breeding—Branch of the L.K. Ernst Federal Research Center for Animal Husbandry, Pushkin, Moskovskoe Shosse, 55a, 196625 St. Petersburg, Russia; olgastan@list.ru (O.S.); Klaus-90@list.ru (N.P.); kurochkin.anton.66@gmail.com (A.K.)

**Keywords:** semen, roosters, cryopreservation, saccharide, trehalose, maltose, medium, molecules number

## Abstract

The combination of saccharides in the composition of a cryopreservation medium may represent a promising method for the preservation of the reproductive cells of male birds. In the current study, cryoprotective media with a combined composition of mono- and di-saccharides were developed. The degree of penetration of reducing saccharide molecules (maltose—Mal20 medium) and non-reducing disaccharide molecules (trehalose—Treh20 medium) from the cryoprotective medium into the cytosol of rooster spermatozoa was studied. LCM control media without disaccharides were used as the control. The number of maltose molecules penetrating from the outside into the cytosol of the spermatozoon was 1.06 × 10^4^, and the number of trehalose molecules was 3.98 × 10^4^. Using a combination of maltose and fructose, the progressive motility of frozen/thawed semen and the fertility rates of eggs were significantly higher ((*p* < 0.05) 40.2% and 68.5%, respectively) than when using a combination of trehalose and fructose in a cryoprotective diluent (33.4% and 62.4%, respectively). A higher rate of chromatin integrity at the level of 92.4% was obtained when using Treh20 versus 74.5% Mal20 (*p* < 0.05). Maltose positively affected the preservation of frozen/thawed sperm in the genital tract of hens. On the seventh day from the last insemination when using Mal20, the fertilization of eggs was 42.6% and only 27.3% when using Treh20. Despite the same molecular weight, maltose and trehalose have different physicochemical and biological properties that determine their function and effectiveness as components of cryoprotective media.

## 1. Introduction

Cryopreservation is a method that allows one to maintain the quality of the biological reproductive material of animals for several years, which is achieved through the careful development of methods and protocols for freezing and thawing. The least successful process is the cryopreservation of semen in poultry. Despite the use of different freezing protocols, taking into account the individual and breed characteristics of chickens, the fertility of eggs remains on average at the level of 30% [1,2,3,4]. However, some researchers have managed to achieve higher fertility rates for frozen/thawed male semen, at the levels of 65% [5] and 82–86% [6], when using the fast protocol.

Damage to reproductive cells that occurs during the freeze/thaw cycle is caused both by the formation of ice crystals and by chemical and physical processes, such as denaturation of proteins and lipids of membrane bilayers, leading to sublethal freezing and the start of the formation of reactive oxygen species [7]. All of these factors cause disruption of the integrity of the membranes of sperm, its organelles, and DNA fragmentation. One of the ways to solve the problem of the cryopreservation of reproductive cells of male birds is the development of protective media with a combination of various endo- and exo-cryoprotectants for maximum preservation of the integrity of spermatozoa and their functional usefulness. When developing a cryoprotective medium, it is necessary to take into account the multifactorial balance of the parameters of osmotic balance, the balance of the sperm enzyme system [8,9], and pH level, and at the same time, provide cryoprotection to stabilize the proteins of the cell membranes and organelles of the sperm. The stability of the protein–lipid structure of cell membranes and their organelles can be ensured through the use of certain saccharides [10]. These include reducing disaccharides, such as maltose, lactose, rabinose, and other and non-reducing disaccharides (for example, trehalose and sucrose). Monosaccharides (D-fructose, D-glucose, and galactose) [11] and carbocyclic sugar inositol [12], which have multidirectional properties (energy supply to cells and maintenance of osmotic balance), are also used in the compositions of cryoprotective media. The molecular weight, composition, structure, and size of the glycosidic bonds of saccharides affect their ability to penetrate cell membranes and determine their cryoprotective ability in the composition of semen diluents for cryopreservation.

For example, the non-reducing saccharide trehalose can act as a kosmotrope and create a closer trehalose–water interaction than a water–water interaction, which determines its bioprotective effect [13], increasing the viscosity of the cytoplasm [14]. Moreover, it is capable of forming in cells of a stable vitreous matrix with extremely low molecular mobility during low-temperature stress [15]. Trehalose increases the stability of various macromolecules through preferential hydration during various physical influences [16]; its role is to stabilize phospholipids upon cooling and prevent disruption of the bilayer structure of cell membranes [17,18]. The ordering of water molecules around trehalose prevents ice formation. Slowing down the rate of protein aggregation with the participation of trehalose prevents their denaturation. Trehalose is able to regulate osmotic pressure and hydration balance in cells depending on changes in the activity of the surrounding water, which protects the cell from osmotic changes during freezing by controlling the activity of water through the formation of long-lived hydrogen bonds. All of these features make trehalose one of the best known cryoprotectants [19,20].

The difference between reducing and non-reducing disaccharides lies in the size of their glycosidic bonds and the ability or inability of their molecules to mutarotate, which determines the interaction of saccharides with the components of the diluent and directly with the organic structures of the cell at various stages of the cryopreservation process. Maltose is a reducing sugar because it has an unsubstituted hemiacetal hydroxyl group; in solutions, maltose can exist in two forms—cyclic and aldehyde, which are in dynamic equilibrium.

The use of reducing saccharides in cryoprotective media is justified by their ability to bind to carbohydrate structures of the cell membrane (glycocalyx) [21], which suggests the possibility of its attachment to the glycocalyx construct—a progressive evolutionary cellular structure that provides the possibility of specific cellular adaptations to certain temperature, chemical, and other paratypical influences. Glycocalyx is a dense carbohydrate layer (20–60 nm) on the surface of the spermatozoa membrane, which is associated with plasma membrane proteins (glycoproteins) and lipids (glycolipids). The glycocalyx of native spermatozoa changes significantly at different stages of their life cycle and represents the “primary interface” between the spermatozoa and the environment. Since the surface carbohydrates of the outer membrane of spermatozoa affect their fertility, the changes in the glycocalyx occurring during the freezing/thawing of spermatozoa affect their viability and motility, as well as the safety and transport in the reproductive tract of the hen [21].

In addition, the maltose molecule can easily break down into two glucose molecules, followed by glycolysis, which makes it a reserve source of available energy for reproductive cells. At the same time, the effectiveness of maltose as a cryoprotective diluent for chicken semen has been little studied, but its potential beneficial effect has been found in other animal species [22,23].

According to the literature, various combinations of non-reducing and reducing saccharides can provide semen with the most effective protection during freezing/thawing [24]. The effect of a combination of monosaccharides (fructose, galactose, glucose, and xylose) and disaccharides (lactose, trehalose, maltose, and sucrose) on the composition of diluents for semen cryopreservation has been evaluated on various animal species [23,25,26].

The aim of our study was to determine the degree of penetration of molecules of the reducing disaccharide maltose and non-reducing disaccharide trehalose into the cytosol of rooster spermatozoa from the cryoprotective medium. The aim of this study was to determine the effect of the combination of mono- and di-saccharides on the composition of the cryoprotective medium and on the maintenance of the functional usefulness of *Gallus domesticus* spermatozoa in the freezing/thawing cycle.

## 2. Results

### 2.1. Results of Evaluation of Sperm Motility, Membrane Integrity, and Chromatin Damage in Native and Frozen/Thawed Semen

Progressive motility of frozen/thawed spermatozoa with Mal20 diluent was significantly higher than with LCM control (*p* < 0.05). The use of Treh20 medium did not affect the progressive sperm activity. The use of experimental diluents Mal20 and Treh20 made it possible to significantly reduce damage to spermatozoa membranes compared with LCM control. Significant differences in the degree of membrane damage of frozen/thawed spermatozoa were observed when using experimental diluents in relation to the control (*p* < 0.05). The level of chromatin integrity was significantly higher when using Treh20 medium compared with LCM control and Mal20 (*p* < 0.05) (Table 1).

### 2.2. Results of Chromatographic Analysis of Carbohydrate Content in Cytosol of Spermatozoa

**Figure 1:**
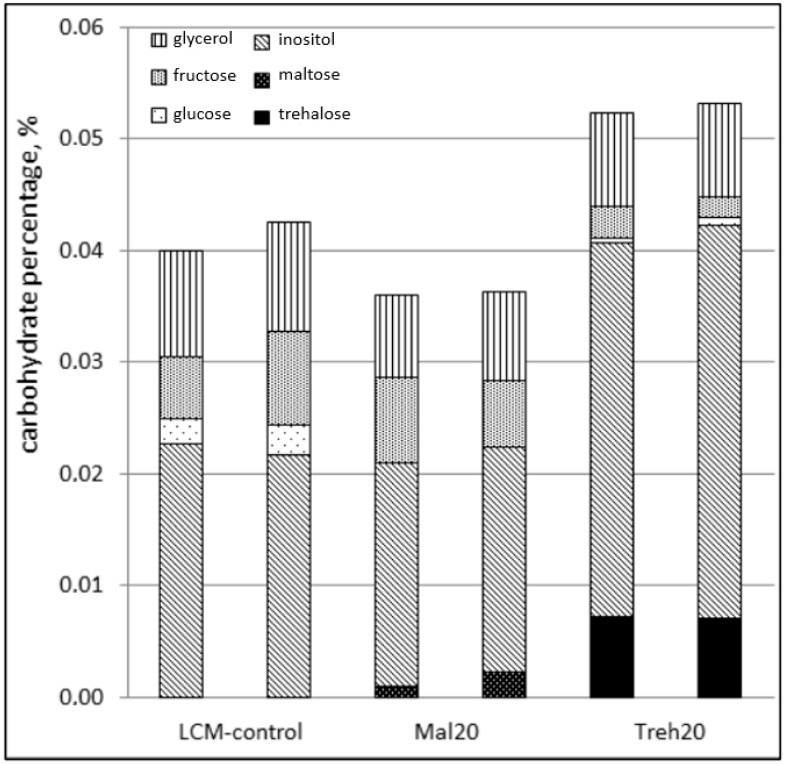
Carbohydrate composition of spermatozoa cytosol in native rooster semen after equilibration with cryoprotective diluents (freezing protocol is presented in Section 4.3; composition of diluents LCM control, Mal20, and Treh20 is presented in Table 5; evaluation of each sample was carried out in duplicate).

Determination of the quantitative composition of carbohydrates (glucose, inositol, trehalose, maltose, and fructose) and glycerol in rooster spermatozoa after the exposure stage included in the semen freezing protocol showed the following results: In the control sample (LCM control), the fructose content averaged 16.7% of the total carbohydrate content in the spermatozoa cytosol in the absence of maltose and trehalose. In the Mal20 sample, the fructose content was 18.7%, the maltose content was 4.4%, and there was no trehalose. In the Treh20 sample, the fructose content was 4.3%, the trehalose content was 13.5%, and maltose was absent (Table 2, Figure 1).

To determine the degree of penetration of saccharides from the cryoprotective diluent into the cytosol of spermatozoa, the number of molecules of the studied saccharides per 1 mL of diluted sperm was theoretically calculated; then, we calculated the number of saccharide molecules inside the cell after equilibration according to the results of chromatographic analysis (Table 3).

Taking into account the average sperm concentration in the ejaculates evaluated in the experiment (~2.2 billion/mL), the theoretical number of saccharide molecules penetrating into the sperm during the equilibration of the semen with cryoprotective diluents was calculated (Table 4).

Despite the seemingly insignificant amount of saccharides penetrating from the outside into the spermatozoa, the number of molecules per cell is fairly large—1.06 × 10^4^ maltose molecules and 3.98 × 10^4^ trehalose molecules. It is noteworthy that with the same external concentration, the content of trehalose in the sperm cell was three times higher than that of maltose.

### 2.3. Fertility Assessment of Frozen/Thawed Semen

**Figure 2:**
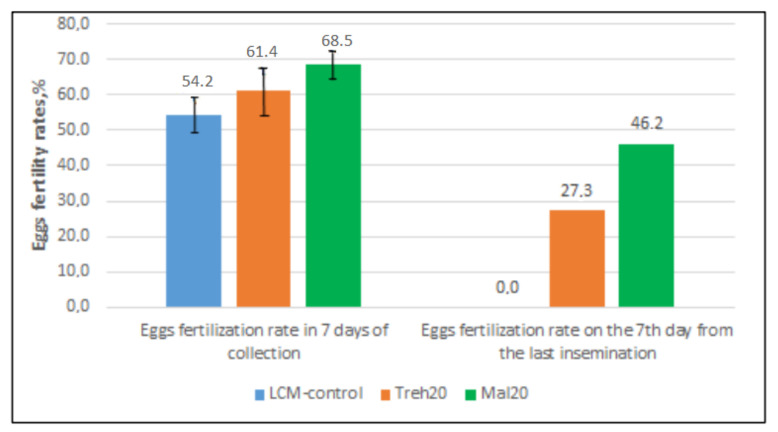
Fertility of eggs of hens inseminated with frozen/thawed semen using cryoprotective diluents LCM control, Treh20, and Mal20. The composition of the diluents LCM control, Mal20, and Treh20 is presented in Table 5.

According to the results of the artificial insemination of hens, significantly higher fertility rates for frozen/thawed semen were obtained with the use of Mal20 and Treh20 diluents containing combinations of mono- and disaccharides compared with LCM control. Significant differences at the level of *p* < 0.05 were obtained for the LCM control and Mal20 groups (Figure 2). The group of hens inseminated with frozen/thawed semen with Mal20 diluent achieved the highest egg fertilization rate of 68.5%. Moreover, in this group, the best rate of the preservation of sperm fertility in the genital tract of hens after 7 days from the last insemination was obtained—46.2%, which is a high result for cryopreserved rooster semen.

**Figure 3:**
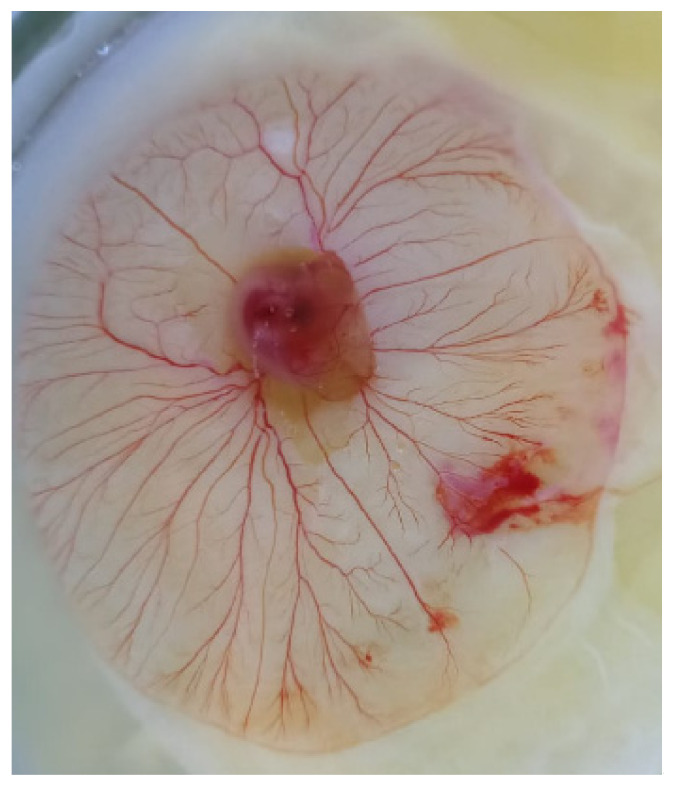
Development of the embryo of the Treh20 experimental group on the 7th day of incubation.

The maximum egg fertility rates (88.9% and 81.3%) were obtained on the fourth and fifth day of egg collection in the group of hens inseminated with frozen/thawed semen using the Treh20 extender. In the group of hens inseminated with frozen/thawed semen using the LCM control diluent, the egg fertilization rates were 75.0% and 55.6% on the same days. Evaluation of the development of embryos on the seventh day of egg incubation showed that the embryos corresponded to the standards of development according to Hamburger and Hamilton (1951) [27] stages 29–30 by 100% in the Treh20 and Mal20 groups (Figure 3) and by 98.4% in the LCM control group.

## 3. Discussion

The results of our studies prove that the use of cryoprotective diluents for cock semen, including combinations of monosaccharides (fructose) and disaccharides (maltose and trehalose), can reliably increase the cryoresistance of the semen in the freeze/thaw cycle according to the fast protocol and preserve its viability and fertility.

Fructose is a natural component of rooster semen that easily penetrates the cell membrane of the spermatozoa, and its addition to the cryoprotective medium in an amount of 0.6–0.8 g per 100 mL of water creates an additional energy reserve to ensure sperm motility in critical conditions and allows for the maintenance of osmolarity at the correct level.

Maltose and trehalose are not found in the semen of native roosters. It can be assumed that certain conditions are required for their interaction with the spermatozoan membrane and diffusion through the membrane pores into the cell. We believe that in our experiments, the penetration of disaccharides into the cytosol of spermatozoa was facilitated by a temperature difference equilibrating from 18 °C to 5 °C for 40 min during the process of equilibration of the semen with a cryoprotective medium. The evaluation of the presence of disaccharides in the cytosol of spermatozoa showed that the molecules of trehalose and maltose from the cryoprotective medium penetrate the cells, but to a different extent. In the theoretical calculation of the number of molecules in spermatozoa, we determined 1.06 × 10^4^ molecules for maltose and 3.98 × 10^4^ for trehalose per cell. Although these values are not great, the effectiveness of disaccharide supplementation is undeniable. Despite the same molecular weight, maltose and trehalose have different physicochemical and biological properties that determine their function and effectiveness as components of cryoprotective media.

When assessing the difference between the number of trehalose and maltose molecules in the cytosol of the spermatozoa, the best result was obtained in favor of trehalose, but when using a combination of maltose and fructose, the progressive motility of frozen/thawed semen and the fertility rates of eggs were significantly higher ((*p* < 0.05) 40.2% and 68.5%, respectively) than when using a combination of trehalose and fructose in a cryoprotective diluent (33.4% and 62.4%, respectively. It is likely that this fact is due to the tendency of maltose, as a reducing saccharide, to bind to the structure of the spermatozoa glycocalyx, thereby increasing the strength of the spermatozoa membrane in the freeze/thaw cycle. Another explanation is also possible. According to our hypothesis, based on the literature, maltose is fermented, easily breaking down into two glucose molecules under the influence of the sperm-specific enzyme GAPDS (glyceraldehyde 3-phosphate dehydrogenase-S) [28], and, as a result, it can provide ATP production for the kinetic apparatus of spermatozoa and increase their mobility. The same opinion is shared by Kosova et al. [29], claiming that the thermoprotective effect of the glycolytic enzyme glyceraldehyde 3-phosphate dehydrogenase (GAPDH), as an enzyme of glycolysis, which serves to break down glucose and to obtain energy and carbon molecules, may not consist of protecting the cell from thermal damage but rather providing the cell with the necessary energy during a temperature shock. Elkina et al. [30] also argues that the enhanced stability of the sperm enzyme (GAPDS) is likely to be important for the efficiency of fertilization, providing the sperm motility during the period of its life. Thus, glycolytic enzymes specific to spermatogenic cells can provide flexible use of substrates (in our case, maltose) and enable spermatozoa to adapt to unexpected conditions in the female reproductive tract [31], which was ultimately reflected in our experiments on the rates of egg fertilization and fertility preservation of frozen/thawed sperm in the genital tract of hens. On the seventh day from the last insemination when using the cryoprotective medium Mal20, the fertilization of eggs was 42.6% and only 27.3% when using the medium Treh20.

Trehalose, a non-reducing disaccharide with a stable water bond, is less susceptible to enzymatic reactions and better ensures the preservation of the internal structures of spermatozoa than maltose, which is confirmed by the results of our experiments—a higher rate of chromatin integrity at the level of 92.4% when using Treh20 media versus 74.5% with Mal20 (*p* < 0.05). The level of chromatin integrity has a significant impact on the results of the incubation of eggs when using frozen/thawed semen for artificial insemination [32]; therefore, the degree of DNA integrity should be considered in the integrated assessment and selection of frozen/thawed semen.

Physicochemical processes occurring in the cell with the participation of trehalose explain its cryoprotective function. The addition of disaccharides to water leads to a drastic reorganization of the hydrogen-bonded network [19]. However, trehalose is capable of changing the structure of bound water to a greater extent than maltose, lowering the freezing point of biological solutions to a lower value and preventing the formation of ice crystals, which are one of the most important damaging factors for the cell during its freezing/thawing [19]. It is generally accepted that the maximum replacement of water in the cell helps to protect it from the formation of ice crystals, but it is also believed that there is a limit to the replacement of water molecules by trehalose, and exceeding this level can provoke a violation of the lipid layer of membranes and thereby cause degradation of the plasma membrane of the cell [33].

In summary, it should be concluded that the use of saccharides as components of cryoprotective media for low-temperature freezing of bird semen has great prospects from the point of view of maintaining the functional usefulness and fertility of frozen/thawed spermatozoa. Research in this direction should be continued, taking into account the physicochemical characteristics and biological function of saccharides, their possible mutual transformations, and their ability to interact with the membranes of spermatozoa.

## 4. Materials and Methods

### 4.1. Animals

The animals used during this study were 20 roosters and 75 hens of the Rhode Island Red breed kept in individual battery cages (size = 45 × 60 × 60 cm, photoperiod = 14 L:10 D, temperature = 18 °C). The experimental population was obtained according to the standards accepted in the Center of Collective Usage “Genetic collection of rare and endangered chicken breeds” of the Russian Research Institute of Farm Animal Genetics and Breeding—Branch of the L.K. Ernst Federal Research Center for Animal Husbandry (RRIFAGB). The experiment was carried out in the period of January–May 2021.

### 4.2. Semen Collection

Semen was collected from Rhode Island Red cocks at age of 48–50 weeks (*n* = 20) twice a week by abdominal massage. Each ejaculate was evaluated individually and selected according to the following criteria: volume (graduated pipette) = 0.2–1.2 mm; sperm concentration (Accurate Photometer, IMV Technologies, Bellshill, UK, 2019) ≥2.0 billion/mL; total and progressive sperm motility using computer-assisted sperm analysis (Motic BA410E, China, 2019, negative contrast, ×200; digital input system BASLER acA1300) and software (ArgusSoft, Saint-Petersburg, Russia, 2020) ≥85%; percentage of agglutination (Motic agglutinationBA410E, China, 2019, negative contrast, ×200, magnification × 100) ≤10%.

### 4.3. Spermatozoa Cryopreservation: Media and Procedure

Semen was frozen in field conditions. The resulting semen was pooled to eliminate individual differences, divided into 3 aliquots, and diluted with cryoprotective media (Table 5) in a 1:1 ratio. Diluted semen samples were equilibrated from 18 °C to 5 °C for 40 min. After cooling, dimethylacetamide (DMA, Sigma Aldrich, St. Louis, MO, USA) was added to each sample at a final concentration of 6%. After adding DMA, the samples were incubated at 5 °C for 1 min. Freezing was carried out in pellets by directly dripping the semen into liquid nitrogen. The starting position of the glass Pasteur pipette with semen was monitored using a digital manual temperature indicator with a sensor (THERM 2420, AHLBORN, Holzkirchen, Germany). In the region where the pipette was placed above the nitrogen surface, the temperature was between −15 °C and −20 °C. The average semen digging rate was ~1.4 pellets per second. The pellets were placed in marked glass vials and stored at −196 °C for 70 days. The pellets were thawed on a heated metal plate at 60 °C (self-developed equipment, RRIFAGB 1989, Saint-Petersburg, Russia).

### 4.4. Plasma Membrane Damage (Sperm Viability)

Sperm viability was examined on the basis of histological smears stained with nigrosin-eosin at 1000-fold magnification (Motic BA410E, China, 2019, with ×1000 immersion magnification). In each smear, the number of dead, morphologically damaged, and unaltered spermatozoa were counted. All pink-stained spermatozoa were considered dead. The results were expressed as the percentage of certain categories of spermatozoa (each sample was estimated at 200 cells, which was taken as 100%) [35,36].

### 4.5. Sperm Chromatin Integrity

The chromatin integrity was assessed for native and frozen/thawed spermatozoa. A drop of semen diluted at a 1:20 ratio for smear preparation was placed on a glass slide and then fixed with an acetic acid solution (one part acetic acid to three parts 96% ethyl alcohol) for 1 h at a temperature of 5 °C. The prepared samples were immersed in the prepared dye (McIlvaine citrate-phosphate buffer (40 mL of citric acid solution, 2.5 mL of sodium hydrogen phosphate solution, pH 2.5) and 10 mL of 1% acridine orange). Then, it was dried in air for 1–2 min. Microscopy was performed using a UV filter with a wavelength spectrum of 530 nm at magnifications of ×300 and ×600 (Leica DM 4000b, Leica Microsystems GmbH, Wetzlar, Germany, St. Petersburg State University). In each sample, at least 200 cells were evaluated. Cells with a gradient from green to orange were counted as cells with intact chromatin [37].

### 4.6. Quantitative Chromatographic Analysis of Cytosol Carbohydrates of Spermatozoa

To prepare samples for the quantitative analysis of carbohydrates bound to the spermatozoa membrane, the collected semen from 20 males were pooled. The total volume of the obtained semen was at least 15 mL, and it was divided into 3 aliquots; diluted with LCM control, Treh20, and Mal20 media at a 1:1 ratio; and exposed for 40 min (mimicking the first step of the semen freezing protocol). Then, the semen was centrifuged for 10 min at 3000 rpm. The supernatant was removed, a 0.9% sodium chloride solution was added, and the mixture was thoroughly stirred and centrifuged again with the same parameters. The procedure was repeated three times. During the next stage, the centrifuged semen was collected in small doses on a nylon filter and precipitated for another 30 min. Prepared samples of centrifugates and supernatants were frozen and stored at −25 °C.

Analysis of soluble carbohydrates of spermatozoa cytosol was carried out. Dry biomass was gravimetrically determined. The extraction of soluble sugars of spermatozoa (300–400 mg of wet weight) was carried out in 5 mL of water at 100 °C for 20 min four times. Proteins were removed from the combined extract [38]. Further purification of the carbohydrate extract from charged compounds was carried out using a combined column with Dowex−1 (acetate form) and Dowex 50W (H^+^) ion exchange resins. Proteins and charged compounds were also removed from the wash water samples. The composition of carbohydrates was determined using gas–liquid chromatography, obtaining trimethylsilyl sugar derivatives from a lyophilized extract [39]. A-methyl-d-mannoside “Merck” (Darmstadt, Germany) was used as an internal standard. Chromatography was carried out on a Kristall 5000.1 gas–liquid chromatograph (ZAO Khromatek, Russia), on a ZB–5 30 m, 0.32 mm, 0.25 μm capillary column (Phenomenex, USA) using a temperature program from 130 to 270 °C at a rate of 5–6 deg/min. Glycerin, glucose, inositol, trehalose, and maltose (Sigma, USA) were used as taps. Evaluation was performed in duplicate.

### 4.7. Theoretical Calculation of the Number of Saccharide Molecules in Cryoprotective Media, Diluted Semen, and Spermatozoa Cytosol

To carry out the calculations, the values of the molecular masses of saccharides, the concentration of solutions, the concentration of spermatozoa, and the results of quantitative chromatographic analysis of carbohydrates in the cytosol of spermatozoa were used.

### 4.8. Artificial Insemination

In the current experiment, we used hens at the age of 58–60 weeks, with 25 hens in each experimental group. Before the start of insemination, to control the absence of semen in the genital tract of the chickens, the eggs were assessed twice with an interval of seven days. To test the cocks’ sperm fertility, the hens were inseminated intravaginally according to the following scheme: two days in a row with a single insemination dose of 0.04–0.07 mL of frozen/thawed semen (insemination dose was at least 70–80 million progressively moving spermatozoa) and then every two days. There was a total number of four insemination days. Insemination time was from 14:00 to 16:00. The collection of eggs for incubation began one day after the first insemination and was carried out according to the scheme (Table 6). Eggs were incubated for six days to assess the fertility of frozen/thawed semen (*n* = 429 eggs).

### 4.9. Ethic Statement

The study was conducted in accordance with the guidelines of the Declaration of Helsinki and in accordance with the Russian “Guidelines for accommodation and care of laboratory animals. Rules for keeping and care of farm animals” (State Standard GOST-34088-2017) dated 12 December 2017.

### 4.10. Statistical Analysis

For statistical data processing, the software applications Excel 2013 (Microsoft, Redmond, WA, USA) and Statistica 7.0 (StatSoft, Tulsa, OK, USA) were used. The data (volume of semen and average sperm concentration) corresponded to the normal Gaussian distribution. Data are presented as mean values ± standard deviation (±SE). The associations between egg fertility rates and the types of medium, membrane damage, and chromatin integrity were evaluated using the Mann–Whitney U test and were considered significant at *p* < 0.05.

## Figures and Tables

**Table 1:** Quality indicators of native and frozen/thawed rooster semen depending on the cryoprotective diluent used.

Quality Indicators	Fresh Semen	Frozen/Thawed Sperm		
		LCM Control *	Mal20 *	Treh20 *
Total motility (TM), %	86.3 ± 0.04	52.7 ± 0.7	55.4 ± 1.5	52.9 ± 0.9
Progressive motility (PM), %	65.7 ± 0.14	34.3 ± 2.6 ^a^	40.2 ± 3.8 ^b^	33.4 ± 2.6 ^a^
Membrane integrity, %	90.4 ± 0.01	48.0 ± 7.7 ^a^	67.2 ± 2.3 ^b^	69.3 ± 2.8 ^b^
Chromatin integrity, %	95.6 ± 0.01	79.5 ± 5.2	74.5 ± 4.1 ^a^	92.4 ± 5.9 ^b^

* Note: The composition of the diluents LCM control, Mal20, and Treh20 is shown in Table 5. ^a,b^ *p* < 0.05.

**Table 2:** Results of the analysis of the quantitative composition of carbohydrates (glucose, inositol, trehalose, maltose, and fructose) and glycerol in the spermatozoa of roosters (in duplicate).

		LCM Control		Mal20		Treh20	
		1	2	1	2	1	2
% of dry biomass	Glycerol	0.010	0.010	0.008	0.008	0.009	0.008
	Fructose	0.005	0.008	0.008	0.006	0.003	0.002
	Glucose	0.002	0.003	0.000	0.000	0.000	0.001
	Inositol	0.022	0.021	0.020	0.020	0.035	0.035
	Maltose	0.000	0.000	0.001	0.002	0.000	0.000
	Trehalose	0.000	0.000	0.000	0.000	0.008	0.007
Σ % of dry biomass		0.040	0.042	0.036	0.036	0.054	0.053
% of Σ	Glycerol	24.3	23.3	21.1	22.3	16.3	16.0
	Fructose	13.8	19.7	21.1	16.3	5.3	3.4
	Glucose	5.6	6.3	0.0	0.0	0.9	1.4
	Inositol	56.3	50.7	55.0	55.2	63.5	65.7
	Maltose	0.0	0.0	2.7	6.1	0.0	0.0
	Trehalose	0.0	0.0	0.0	0.0	14.0	13.5

**Table 3:** Number of molecules in the media and the number of molecules introduced into the native semen after equilibration (dilution 1:1), pcs/mL (theoretical calculation).

Saccharide	LCM Control *		Mal20 *		Treh20 *	
	The Number of Molecules					
	In the Media (per 1 mL)	Introduced into the Native Semen (per 1 mL)	In the Media (per 1 mL)	Introduced into the Native Semen (per 1 mL)	In the Media (per 1 mL)	Introduced into the Native Semen (per 1 mL)
Fructose	0.265 × 10^20^	1.463 × 10^14^	0.216 × 10^20^	0.517 × 10^14^	0.216 × 10^20^	0.675 × 10^14^
Maltose	−	−	0.057 × 10^20^	0.233 × 10^14^	−	−
Trehalose	−	−	−	−	0.057 × 10^20^	0.875 × 10^14^

* Note: The composition of the diluents LCM control, Mal20, and Treh20 is shown in Table 5.

**Table 4:** Theoretical number of molecules in the cytosol of each spermatozoa after equilibration for 40 min, pcs.

Saccharide	The Number of Molecules in the Cytosol of the Spermatozoa after Equilibration, pcs		
	LCM Control *	Mal20 *	Treh20 *
Fructose	6.65 × 10^4^	2.35 × 10^4^	3.07 × 10^4^
Maltose	−	1.06 × 10^4^	−
Trehalose	−	−	3.98 × 10^4^

* Note: The composition of the diluents LCM control, Mal20, and Treh20 is shown in Table 5.

**Table 5:** Composition of media for freezing the semen of roosters.

Medium Composition	Medium		
	LCM Control *	Treh20 *	Mal20 *
Monosodium glutamate	1.92 g (114 mM)	1.92 g (114 mM)	1.92 g (114 mM)
Fructose	0.8 g (44 mM)	0.64 g (36 mM)	0.64 g (36 mM)
Potassium acetate	0.5 g (51 mM)	0.5 g (5 mM)	0.5 g (5 mM)
Polyvinylpyrrolidone	0.3 g (8.3 mM)	0.3 g (8.3 mM)	0.3 g (8.3 mM)
Protamine sulfate	0.032 g (3.27 mM)	0.032 g (3.27 mM)	0.032 g (3.27 mM)
Trehalose	−	0.326 g (9.5 mM)	−
Maltose	−	−	0.326 g (9.5 mM)
Distilled water	100 mL		
Osmolarity	339 mOsm	344 mOsm	334 mOsm

* Note: Leningrad cryoprotective medium (LCM) for semen freezing [6,34].

**Table 6:** Scheme for hen insemination, collection of eggs for incubation, and collection of eggs to assess the survivability of spermatozoa in hens’ genital tract.

	Day of Experiment														
	1st	2nd	3rd	4th	5th	6th	7th	8th	9th	10th	11th	12th	13th	14th	15th
Insemination	+	+			+			+							
Collecting eggs for 1st incubation			+	+	+	+	+	+	+						
Collecting eggs for 2nd incubation															+

## Data Availability

Not applicable.

## References

[1] Seigneurin F., Blesbois E. (2010). Update on Semen Cryopreservation Methods in Poultry Species. 13th European Poultry Conference (EPC 2010).

[2] Long J.A., Bongalhardo D.C., Pelaez J., Saxena S., Settar P., O’Sullivan N.P., Fulton J.E. (2010). Rooster semen cryopreservation: Effect of pedigree line and male age on post-thaw sperm function. Poult. Sci..

[3] Ciftci Y., Aygun A. (2018). Poultry semen cryopreservation technologies. Worlds Poult. Sci. J..

[4] Fulton J.E. (2006). Avian genetic stock preservation: An industry perspective. Poult. Sci..

[5] Silyukova Y., Pleshanov N., Stanishevskaya O. (2019). The influence membranes damage and activity of roosters’ sperm on the fertilization of eggs when using cured cryopreserved sperm. Reprod. Domest. Anim..

[6] Stanishevskaya O., Silyukova Y., Pleshanov N., Kurochkin A., Fedorova E., Fedorova Z., Perinek O., Prituzhalova A., Meftakh I. (2021). Effects of Saccharides Supplementation in the Extender of Cryopreserved Rooster (*Gallus domesticus*) Semen on the Fertility of Frozen/Thawed Spermatozoa. Animals.

[7] Pini T., Leahy T., Graaf S. (2018). Sublethal sperm freezing damage: Manifestations and solutions. Theriogenology.

[8] Mavrodina T.G., Stanishevskaya O.I., Cherepenov S.V., Silyukova Y.L. (2018). Influence of sperm quality (cryopreserved and native) on the duration of spermatozoa storage in reproductive tracts of turkeys. Anim. Reprod. Sci..

[9] Smith A.M.J., Bonato M., Dzama K., Malecki I.A., Cloete S.W.P. (2018). Mineral profiling of Ostrich (Struthio camelus) seminal plasma and its relationship with semen traits and collection day. Anim. Reprod. Sci..

[10] Tian Y., Visser J.C., Klever J.S., Woerdenbag H.J., Frijlink H.W., Hinrichs W.L.J. (2018). Orodispersible films based on blends of trehalose and pullulan for protein delivery. Eur. J. Pharm. Biopharm..

[11] Gómez-Fernández J., Gómez-Izquierdo E., Tomás C., Mocé E., Mercado E. (2012). Effect of different monosaccharides and disaccharides on boar sperm quality after cryopreservation. Anim. Reprod. Sci..

[12] Saleh R., Assaf H., Abd W., Maged E., Fawzy M., Elsuity M.A. (2017). Positive effects of in-vitro Myo-inositol supplementation of cryopreserved human sperm on the outcome of cryopreservation: A randomized controlled trial. Fertil. Steril..

[13] Branca C., Maccarrone S., Magazù S., Maisano G., Bennington S.M., Taylor J. (2005). Tetrahedral order in homologous disaccharide-water mixtures. J. Chem. Phys..

[14] Kilburn D., Townrow S., Meunier V., Richardson R., Alam A., Ubbink J. (2006). Organization and mobility of water in amorphous and crystalline trehalose. Nat. Mater..

[15] Rabbani G., Choi I. (2018). Roles of osmolytes in protein folding and aggregation in cells and their biotechnological applications. Int. J. Biol. Macromol..

[16] Rao W., Huang H., Wang H., Zhao S., Dumbleton J., Zhao G., He X. (2015). Nanoparticle-Mediated Intracellular Delivery Enables Cryopreservation of Human Adipose-Derived Stem Cells Using Trehalose as the Sole Cryoprotectant. ACS Appl. Mater. Interfaces.

[17] Feofilova E.P., Mysyakina I.S., Usov A.I., Kochkina G.A. (2014). Trehalose: Chemical structure, biological functions, and practical application. Microbiology.

[18] Rudolph A.S., Crowe J.H., Crowe L.M. (1986). Effects of three stabilizing agents—Proline, betaine, and trehalose—On membrane phospholipids. Arch. Biochem. Biophys..

[19] Jain N.K., Roy I. (2009). Effect of trehalose on protein structure. Protein Sci..

[20] Choi Y., Cho K.W., Jeong K., Jung S. (2006). Molecular dynamics simulations of trehalose as a “dynamic reducer” for solvent water molecules in the hydration shell. Carbohydr. Res..

[21] Peláez J., Bongalhardo D.C., Long J.A. (2011). Characterizing the glycocalyx of poultry spermatozoa: III. Semen cryopreservation methods alter the carbohydrate component of rooster sperm membrane glycoconjugates. Poult. Sci..

[22] Yildiz C., Kaya A., Aksoy M., Tekeli T. (2000). Influence of sugar supplementation of the extender on motility, viability and acrosomal integrity of dog spermatozoa during freezing. Theriogenology.

[23] Golshahi K., Aramli M.S., Nazari R.M., Habibi H. (2018). Disaccharide supplementation of extenders is an effective means of improving the cryopreservation of semen in sturgeon. Aquaculture.

[24] Xi M.D., Li P., Du H., Qiao X.M., Liu Z.G., Wei Q.W. (2018). Disaccharide combinations and the expression of enolase3 and plasma membrane Ca^2+^ ATPase isoform in sturgeon sperm cryopreservation. Reprod. Domest. Anim..

[25] Naing S.W., Wahid H., Mohd Azam K., Rosnina Y., Zuki A.B., Kazhal S., San M.M. (2010). Effect of sugars on characteristics of Boer goat semen after cryopreservation. Anim. Reprod. Sci..

[26] Fernández-Santos M.R., Martínez-Pastor F., García-Macías V., Esteso M.C., Soler A.J., de Paz P., Garde J.J. (2007). Extender osmolality and sugar supplementation exert a complex effect on the cryopreservation of Iberian red deer (Cervus elaphus hispanicus) epididymal spermatozoa. Theriogenology.

[27] Hamburger V., Hamilton H.L. (1951). A series of normal stages in the development of the chick embryo. J. Morphol..

[28] Feiden S., Wolfrum U., Wegener G., Kamp G. (2008). Expression and compartmentalisation of the glycolytic enzymes GAPDH and pyruvate kinase in boar spermatogenesis. Reprod. Fertil. Dev..

[29] Kosova A.A., Khodyreva S.N., Lavrik O.I. (2017). Role of glyceraldehyde-3-phosphate dehydrogenase (GAPDH) in DNA repair. Biochemistry.

[30] Elkina Y.L., Kuravsky M.L., El’darov M.A., Stogov S.V., Muronetz V.I., Schmalhausen E.V. (2010). Recombinant human sperm-specific glyceraldehyde-3-phosphate dehydrogenase: Structural basis for enhanced stability. Biochim. Biophys. Acta.

[31] Miki K. (2007). Energy metabolism and sperm function. Soc. Reprod. Fertil. Suppl..

[32] Salehi M., Mahdavi A.H., Sharafi M., Shahverdi A. (2020). Cryopreservation of rooster semen: Evidence for the epigenetic modifications of thawed sperm. Theriogenology.

[33] Chen T., Acker J.P., Eroglu A., Cheley S., Bayley H., Fowler A., Toner M. (2001). Beneficial Effect of Intracellular Trehalose on the Membrane Integrity of Dried Mammalian Cells. Cryobiology.

[34] Silyukova Y.I., Stanishevskaya O.I., Pleshanov N.V., Kurochkin A.A. (2020). Efficiency of using a combination of mono- and disac-charides in a diluent for freezing rooster semen. Sel’skokhozyaistvennaya Biol..

[35] Pintado B., de la Fuente J., Roldan E.R.S. (2000). Permeability of boar and bull spermatozoa to the nucleic acid stains propidium iodide or Hoechst 33258, or to eosin: Accuracy in the assessment of cell viability. Journal of reproduction and fertility. J. Reprod. Fertil..

[36] Banaszewska D., Andraszek K., Zdrowowicz E., Czubaszek M., Walczak-Jędrzejowska R. (2015). The role of staining techniques in seminological analysis of mammalian semen. Folia Pomeranae Univ. Technol. Stetin. Agric. Aliment. Piscaria Zootech..

[37] Kazerooni T., Asadi N., Jadid L., Kazerooni M., Ghanadi A., Ghaffarpasand F., Kazerooni Y., Zolghadr J. (2009). Evaluation of sperm’s chromatin quality with acridine orange test, chromomycin A3 and aniline blue staining in couples with unexplained recurrent abortion. J. Assist. Reprod. Genet..

[38] Somogui M. (1945). Determination of blood sugar. J. Biol. Chem..

[39] Brobst K.M., Scobell H.D. (1982). Modern Chromatographic Methods for the Analysis of Carbohydrate Mixtures. Starch-Stärke.

